# A Diagnosis of Exclusion: Unraveling Adult-Onset Still's Disease

**DOI:** 10.7759/cureus.106732

**Published:** 2026-04-09

**Authors:** Anjiya Aswani, Oluwaseyi A Akinyooye, Mark Gudger, Claudia Barreda-Velit, Olga Abreu, Shaunique Ferguson, Chelsea Thompson

**Affiliations:** 1 Internal Medicine, Ross University School of Medicine, Chicago, USA; 2 Obstetrics and Gynecology, Ross University School of Medicine, Chicago, USA; 3 Family Medicine, Mount Sinai Hospital, Chicago, USA; 4 Rheumatology, Sinai Medical Group, Chicago, USA

**Keywords:** adult-onset still’s disease (aosd), autoinflammatory disorder, macrophage activation syndrome (mas), polyarthralgia, quotidian fever

## Abstract

Adult-onset Still's disease (AOSD) is a rare systemic autoinflammatory disorder characterized by quotidian fevers, arthralgia, transient rash, and elevated inflammatory markers, often leading to diagnostic delay due to overlap with infectious, autoimmune, and malignant conditions. We present the case of a 60-year-old Latina woman who developed daily fevers, polyarthralgia, rash, and hypoxemic respiratory failure. Despite broad-spectrum antibiotics, her symptoms persisted, and extensive infectious and hematologic evaluations were unrevealing. A diagnosis of AOSD was established based on the Yamaguchi criteria and markedly elevated ferritin. The patient responded to corticosteroids but later developed macrophage activation syndrome (MAS), requiring anakinra in addition to high-dose steroids. This case highlights the diagnostic challenges of AOSD in older adults, the potential for atypical systemic manifestations such as pulmonary nodules and anasarca, and the importance of early cytokine-directed therapy to prevent life-threatening complications.

## Introduction

Adult-onset Still's disease (AOSD) is a rare systemic autoinflammatory disorder of unknown etiology, characterized by quotidian fevers, polyarthralgia, transient maculopapular pruritic rash, sore throat, and elevated inflammatory markers [1,2]. Considered the adult counterpart of systemic juvenile idiopathic arthritis, AOSD typically affects individuals between 16 and 35 years, with a slight female predominance, and has an estimated incidence of about 0.16-0.4 cases per 100,000 people per year [3].

Although its pathogenesis remains unclear, dysregulated activation of innate immunity and cytokines, such as interleukin-1 (IL-1), IL-6, and IL-18, are believed to play central roles [4,5]. Environmental triggers can provoke an abnormal immune response via toll-like receptors (TLRs) [5]. The major function of these receptors is the detection of damage-associated molecular patterns (DAMPs) and pathogen-associated molecular patterns (PAMPs), recruiting neutrophils and activating inflammation [5]. As reported in some studies, over-activation of neutrophils (neutrophilia) can occur in over 80% of patients [5]. This can come about as neutrophils release excessive extracellular traps (NETs), a form of neutrophil-death, thereby further activating pro-inflammatory macrophages [5]. This cascade establishes a cycle of inflammation where activated macrophages, marked by elevated serum levels of macrophage migration inhibitory factor (MIF) and macrophage stimulating factor (M-CSF), are elevated, advancing the disease [5].

The resulting dysregulation leads to an upregulation of pro-inflammatory cytokines, such as TNF-α, IL-1β, IL-6, and IL-18 [5]. These play a key role in the pathogenesis of AOSD, and the serum cytokine profiles of patients often show an overexpression of these cytokines [5]. TNF-α, produced by macrophages, contributes to joint damage and instability, and systemic symptoms such as cachexia [5]. IL-1β and IL-6 are secreted by innate immune cells to exert inflammatory effects and are elevated in the serum, lymph nodes, skin, and synovial membranes [5]. Elevated levels of IL-6 are strongly correlated with disease activity and induction of acute-phase reactants, such as C-reactive protein (CRP) and serum amyloid (SAA) [5]. This directly contributes to clinical features, such as fever, arthritis, and the abnormal lab results characteristic of AOSD [5].

Due to its nonspecific clinical features, AOSD is often misdiagnosed or mistaken for infections, autoimmune diseases, or malignancies [6]. Diagnosis relies primarily on clinical criteria, such as the Yamaguchi or Fautrel sets, combined with exclusion of other disorders [3,7]. Markedly elevated serum ferritin is a well-recognized laboratory clue [8], but no single laboratory or imaging modality confirms the diagnosis.

We present a case of AOSD in a 60-year-old Latina woman with fever, rash, polyarthritis, and respiratory failure. This case highlights the challenges of diagnosing AOSD in older adults and emphasizes the importance of early recognition to prevent complications such as macrophage activation syndrome (MAS).

## Case presentation

A 60-year-old Latina woman with a past medical history of hypertension, type 2 diabetes mellitus with retinopathy, hyperlipidemia, and atrial septal defect presented to a community hospital in Chicago with three weeks of daily fever, cough, body aches, and worsening joint pain. She reported progressive bilateral knee swelling, which limited ambulation. Prior to illness, she was fully independent. She denied alcohol or drug use, recent travel, incarceration, or homelessness.

On admission, vital signs were as follows: temperature of 97.9°F, pulse of 71 bpm, respiratory rate of 21 breaths per minute, and blood pressure of 107/44 mmHg. All values were within normal limits, except for the blood pressure, which was mildly hypotensive. Examination noted fatigue, myalgias, a systolic murmur best heard in the pulmonic area, trace knee effusions, and tenderness. Initial labs showed leukocytosis, anemia, elevated ESR and CRP, and normal renal function (Table 1). Imaging revealed trace knee effusions and no pulmonary consolidation. She was admitted for presumed sepsis, and empiric ceftriaxone and vancomycin were started.

**Table 1:** Summary of the initial laboratory investigations with reference ranges. ALT: alanine transaminase; AST: aspartate aminotransferase; eGFR: estimated glomerular filtration rate

Lab Values	Patient Value	Normal Range
Corrected sodium, mEq/L	128	135-145
Glucose, mg/dL	310	70-99
Corrected calcium, mg/dL	8.9	8.5-10.5
Albumin, g/dL	3.1	3.5-5.0
White blood cell count, ×10³/μL	22.4	4.0-11.0
Hemoglobin, g/dL	9.0	12.0-16.0
Hematocrit, %	26.3	36-46
Platelet count, ×10³/μL	516	150-450
Ferritin, ng/mL	>7500	15-150
C-reactive protein, mg/dL	11.3	<1.0
AST, U/L	25	10-40
ALT, U/L	13	7-56
Creatinine, mg/dL	0.81	0.6-1.2
eGFR, mL/min/1.73 m²	84	>60

**Figure 1:**
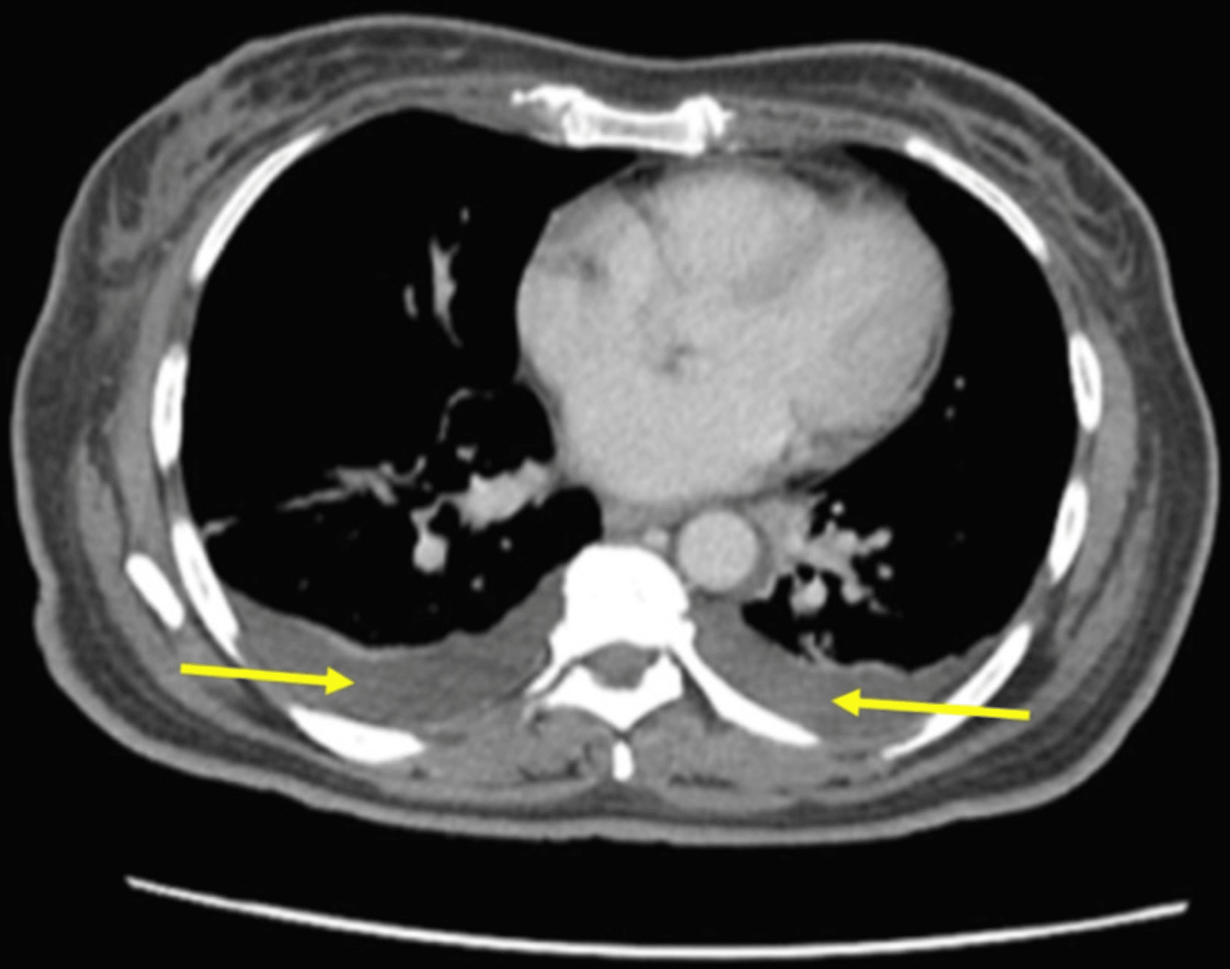
Contrast-enhanced CT of the abdomen and pelvis demonstrating third spacing of fluid with soft tissue anasarca (serious complication of AOSD) and small bilateral pleural effusions (denoted by the yellow arrows). AOSD: adult-onset Still's disease

During hospitalization, she developed persistent fevers up to 39.9°C, worsening polyarthritis, and a pruritic maculopapular rash. Subsequent chest CT revealed multiple small pulmonary nodules, mild pleural effusions, and right axillary lymphadenopathy. Abdominal CT showed anasarca (Figure 1) and mild splenomegaly. She developed acute kidney injury (creatinine of 1.7 mg/dL) and hypoxemic respiratory failure requiring supplemental oxygen.

**Figure 2:**
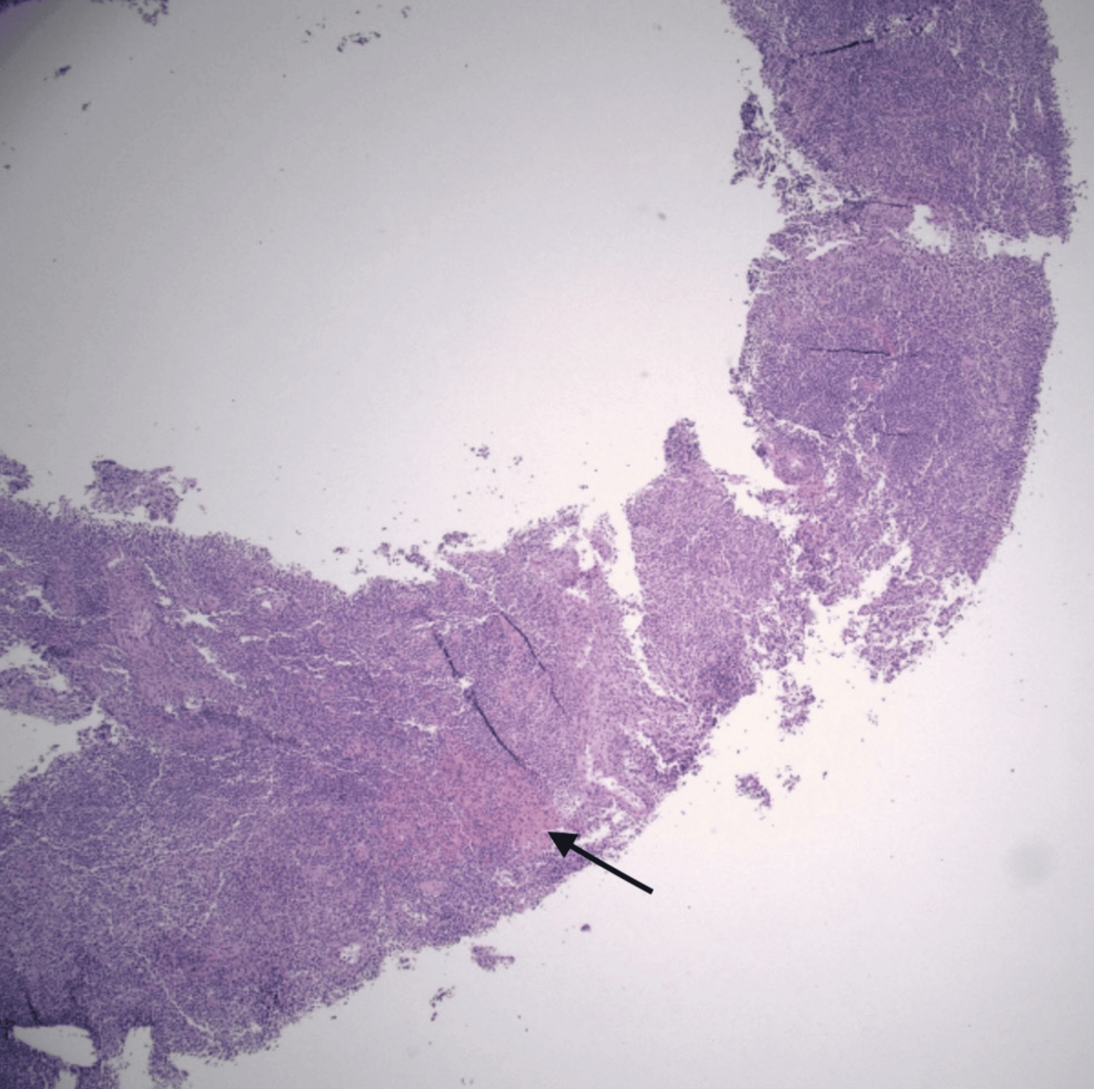
Low-power hematoxylin and eosin (H&E) stain of the lymph node biopsy. Sample shows no evidence of malignancy but displays vascular proliferation, as evidenced by the black arrow.

**Figure 3:**
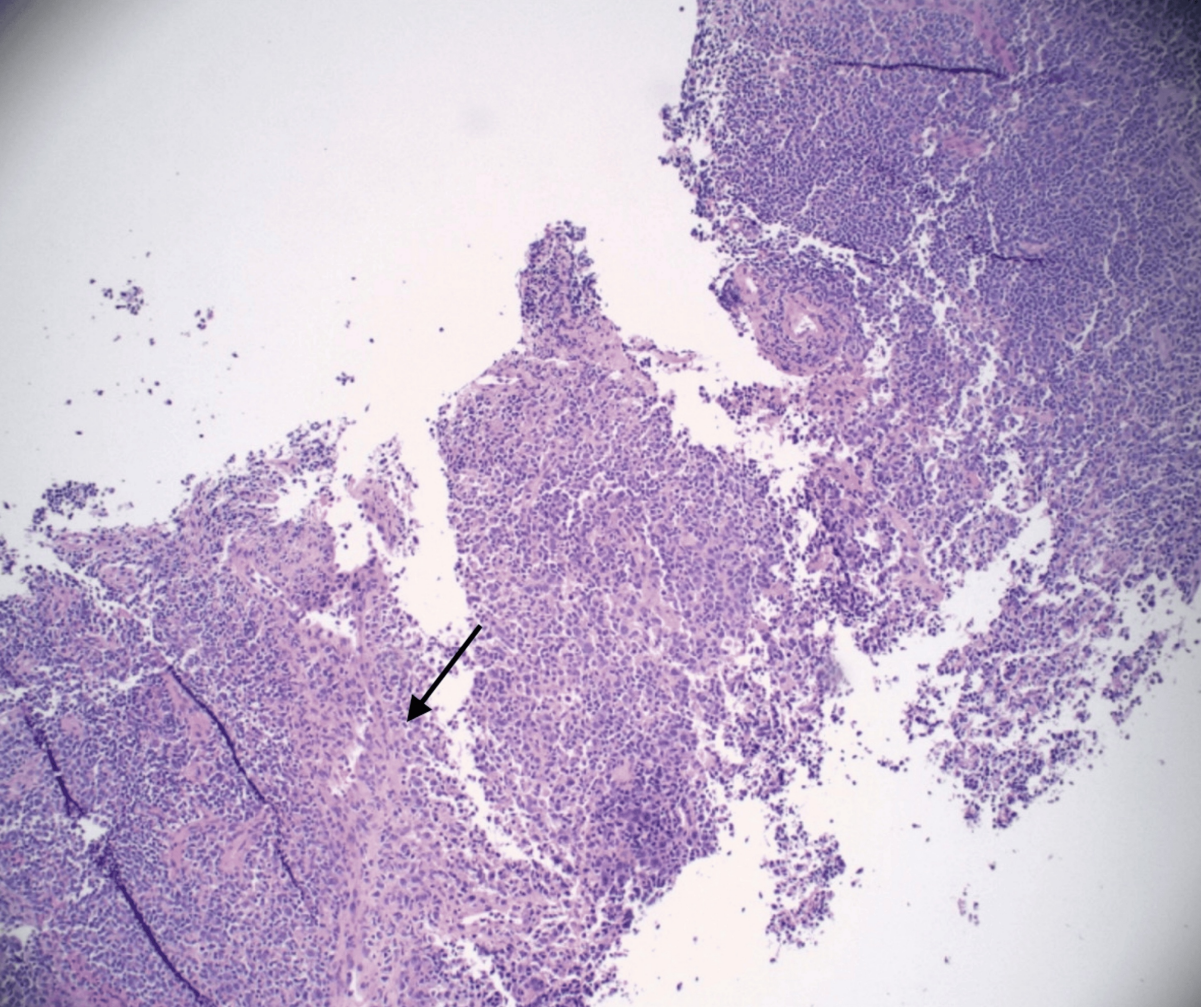
High-power hematoxylin and eosin (H&E) stain of the lymph node biopsy showing vascular proliferation (black arrow).

**Figure 4:**
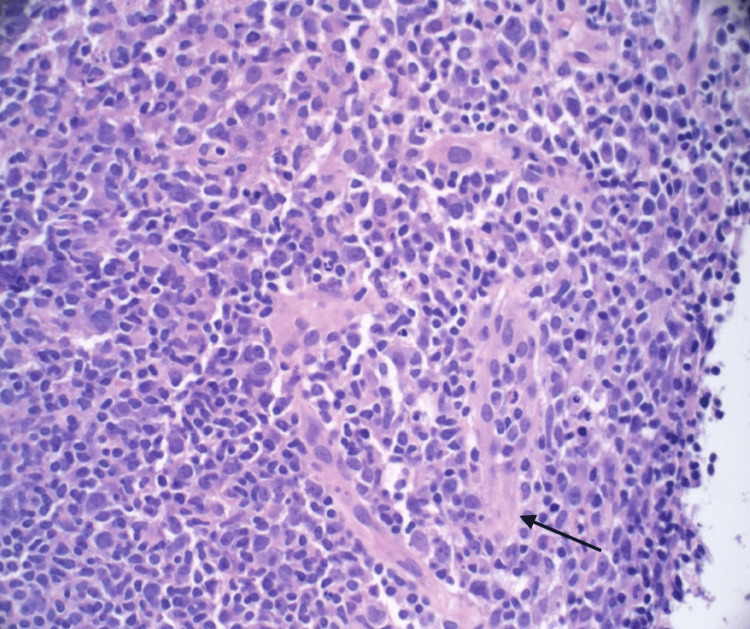
Highest-power hematoxylin and eosin (H&E) stain of the lymph node biopsy showing vascular proliferation (black arrow).

Extensive infectious workup, including HIV, blood cultures, knee aspiration, and lymph node biopsy, was negative for bacterial, viral, or malignant causes (Table 2). Nephrology attributed acute kidney injury to contrast nephropathy and nonsteroidal anti-inflammatory drug (NSAID) exposure. Orthopedic evaluation found no septic arthritis. Hematology ruled out lymphoma after biopsy showed reactive hyperplasia (Figures 2-4).

**Table 2:** Summary of cultures and biopsy performed to rule out other causes.

Laboratory Test	Patient Result	Reference/Normal Range
Blood culture #1	No growth in 5 days	No growth
Blood culture #2	No growth in 5 days	No growth
Left knee fluid culture #1	No growth in 4 days	No growth
Left knee fluid culture #2	No growth in 4 days	No growth
T-cell clonality panel (PCR) – TCRG	Negative	Negative
T-cell clonality panel (PCR) – TCRB	Negative	Negative
HIV test	Negative	Negative

Rheumatology was consulted for persistent fever, rash, and arthritis. Based on Yamaguchi criteria - fever >1 week, arthralgia >2 weeks, leukocytosis with neutrophilia, sore throat, lymphadenopathy, and negative antinuclear antibody (ANA) and rheumatoid factor (RF) - the patient was diagnosed with AOSD [7]. She was started on intravenous methylprednisolone (1 mg/kg), leading to rapid improvement in fever, rash, joint pain, and respiratory status. She was transitioned to oral prednisone (60 mg daily taper), with a plan for outpatient initiation of anakinra.

Two weeks later, she was readmitted to a tertiary center with altered mental status, hypoglycemia, pancytopenia, ferritin >24,000 ng/mL, elevated CRP, and hypertriglyceridemia (263 mg/dL), findings consistent with MAS. Notably, her fibrinogen was markedly elevated (>7,500 mg/dL), a pattern that can be seen in the early inflammatory phase of MAS before levels decline with disease progression. She was treated with pulse intravenous methylprednisolone for three days and intravenous anakinra 100 mg twice daily, with significant improvement. Bone marrow biopsy ruled out malignancy. She was discharged on oral prednisone and subcutaneous anakinra, an IL-1 receptor antagonist that prevents IL-1 from binding to its receptor.

At two-month follow-up, she reported resolution of arthralgia, tapering of corticosteroids, and normalization of inflammatory markers (ferritin decreased from >7,500 ng/mL to 358 ng/mL; CRP negative, Table 3).

**Table 3:** Summary of ferritin and CRP.

Laboratory Test	Initial Value	Most Recent Value	Reference Range
Ferritin	>7,500 ng/mL	358 ng/mL	13-150 ng/mL (female); 30-400 ng/mL (male)
C-Reactive Protein (CRP)	Negative	-	<0.5 mg/dL (negative)

## Discussion

AOSD is rare, with an estimated annual incidence of 0.16-0.4 cases per 100,000 persons [9,10]. The disease typically presents in young adults, but a smaller peak occurs after age 50 [10]. Women are slightly more affected than men [11]. Our patient represents an atypical presentation due to age and comorbidities.

The classic triad of AOSD - fever, arthritis, and rash - is present in 75-95% of patients [12]. Sore throat, myalgia, lymphadenopathy, and splenomegaly are also common [12]. Our patient initially lacked the full triad, which developed during hospitalization. She also presented with atypical features, including pulmonary nodules, anasarca, and hypoxemic respiratory failure, complicating recognition.

Elevated ferritin is a hallmark feature, often exceeding five times normal values, and strongly supports the diagnosis [8]. In our patient, ferritin was markedly elevated during MAS (>24,000 ng/mL).

Similar cases in the literature highlight both classic and atypical presentations. A 78-year-old woman in Nepal presented with fever, rash, and arthritis, initially misdiagnosed as neurotuberculosis. She had ferritin >21,000 ng/mL, with rapid improvement on steroids [8]. Risal et al. described pulmonary nodules and pleural effusions in AOSD, resolving after corticosteroid therapy [13]. Gerfaud-Valentin et al. reported organizing pneumonia causing respiratory failure in AOSD, responsive to steroids [14]. A 36-year-old Hispanic man in Pennsylvania presented with fever and cough, later developing myopericarditis and shock, eventually improving with steroids [15]. These cases, like ours, underscore the importance of considering AOSD in atypical systemic or respiratory presentations.

Complications such as MAS are potentially life-threatening. Risk factors include persistent fever, hepatosplenomegaly, cytopenias, elevated liver enzymes, and hyperferritinemia >10,000 ng/mL [16]. Our patient developed MAS, fitting these risk factors. Diagnostic frameworks, such as Hemophagocytic Lymphohistiocytosis 2004 (HLH-2004) [17] and the 2016 European League Against Rheumatism (EULAR)/American College of Rheumatology (ACR)/Paediatric Rheumatology International Trials Organisation (PRINTO) MAS criteria [18], have been applied in adults.

Management centers on glucocorticoids, often with steroid-sparing agents. The 2022 EULAR and Pediatric Rheumatology European Society (PReS) guidelines recommend early use of IL-1 or IL-6 inhibitors alongside steroids [19]. Anakinra (IL-1 antagonist) and tocilizumab (monoclonal antibody against IL-6 receptor) have demonstrated rapid disease control and glucocorticoid-sparing benefits [20,21]. Our patient responded well to corticosteroids and anakinra, consistent with these recommendations.

Outcomes and prognosis remain variable. Some patients experience a monophasic course, while others develop chronic articular or systemic patterns. MAS is associated with mortality rates up to 10% [22]. Our patient recovered after timely escalation of therapy, underscoring the importance of early recognition and multidisciplinary care.

## Conclusions

AOSD is a rare systemic inflammatory disease with protean manifestations. Diagnosis requires exclusion of infections, malignancies, and autoimmune disorders. Our case illustrates the diagnostic complexity of AOSD in an older adult with atypical features, including pulmonary nodules, anasarca, and MAS. Early recognition, multidisciplinary collaboration, and prompt initiation of corticosteroids with cytokine-directed therapies, such as anakinra, are essential to improve outcomes and prevent complications.
